# Current food labelling practices in online supermarkets in Australia

**DOI:** 10.1186/s12966-023-01504-3

**Published:** 2023-09-26

**Authors:** Damian Maganja, Tazman Davies, Laura Sanavio, Jimmy C. Y. Louie, Mark D. Huffman, Kathy Trieu, Jason H. Y. Wu

**Affiliations:** 1 The George Institute for Global Health, The University of New South Wales, Level 5, 1 King St, Newtown, Sydney, NSW 2042 Australia; 2 University of California, Berkeley, CA 94720 USA; 3 Department of Nursing and Allied Health, School of Health Sciences, Swinburne University of Technology, John St, Hawthorn, VIC 3122 Australia; 4 Global Health Center, Cardiovascular Division, Washington University in St. Louis, 660 S. Euclid Ave, St. Louis, MO 63110 USA; 5 School of Population Health, The University of New South Wales, Samuels Building, Samuel Terry Ave, Kensington, NSW 2033 Australia

**Keywords:** Online shopping, Food environments, Food labelling

## Abstract

**Background:**

Food product labelling can support consumer decision-making. Several food product labels (nutrition information panels (NIPs), ingredients lists, allergen declarations and country-of-origin) are mandated for physical product packaging in Australia, with a voluntary front-of-pack nutrition labelling system, Health Star Ratings (HSRs), also available. However, labelling requirements are not explicitly extended to online settings and the extent to which this information is available in these increasingly important food environments has not been assessed.

**Methods:**

Data from all individual food product pages was collected from the online stores of the two dominant supermarket retailers in Australia using automated web scraping in April–May 2022 (*n* = 22,077 products collected). We assessed the proportion of pages displaying NIPs, ingredients, allergens, country-of-origin and HSRs after excluding products ineligible to display the respective label. We also assessed whether HSRs were differentially available for higher- (healthier) and lower-scoring (less healthy) products, with HSR scores drawn from a comprehensive Australian food composition database, FoodSwitch. A manual inspection of randomly selected product pages (*n* = 100 for each label type per supermarket), drawn from products displaying the relevant label, was conducted to assess whether the labels were immediately visible to users (i.e. without scrolling or clicking). Differences in labelling prevalence and visibility were compared using chi-squared tests.

**Results:**

Across both supermarkets, country-of-origin labelling was almost complete (displayed on 93% of food product pages), but NIPs (49%), ingredients (34%) and allergens (53%) were less frequently displayed. HSRs were infrequently displayed (14% across both supermarkets) and more likely to be applied to higher-scoring products (22% on products with ≥ 3.5HSR v 0.4% on products with < 3.5HSR, p < 0.001). One supermarket was far more likely to make NIPs (100% v 2%, p < 0.001), ingredients (100% v 19%, p < 0.001) and allergens (97% v 0%, p < 0.001) information immediately visible, though the other made HSRs more apparent (22% v 75%, p < 0.001). Both supermarkets displayed country-of-origin labels prominently (100% v 86%, p < 0.001).

**Conclusions:**

Food product labelling varies in online supermarkets in Australia overall and between supermarkets, while the design of online stores resulted in differences in labelling visibility. The near-complete display of country-of-origin labels and differential application of HSRs to higher-scoring products may reflect their use as marketing tools. Our findings highlight an urgent need for food labelling regulations to be updated to better account for online retail food environments.

**Supplementary Information:**

The online version contains supplementary material available at 10.1186/s12966-023-01504-3.

## Background

Food product labelling, encompassing a range of different formats and purposes, has the potential to promote healthier dietary patterns and is recommended by the World Health Organization [1]. This involves the display of information on or near products for sale which provides an insight into product composition or other attributes to provide consumers with information to support decision-making.

Though the specific formats and requirements differ from country to country, there are a number of common labelling types [1]. Labels which are typically mandated include quantitative nutrient declarations [2], ingredients lists [3], allergen declarations [3], and country-of-origin labelling [3]. Voluntary forms include front of pack (FOP) nutrition labelling [2, 3] and health and nutrition claims [3, 4].

Australian regulation around product labelling is generally aligned with Codex Alimentarius requirements, with detailed nutrition information (in the form of a nutrition information panel (NIP)) [5], ingredient and allergen declarations [6, 7] and country-of-origin labelling [8] mandated on-pack for most retail packaged products. A government-led but voluntary FOP nutrition label (Health Star Ratings (HSR)) [9] and voluntary health and nutrition claims are also available [10].

While labelling requirements are clear for physical food products, there is less clarity about labelling requirements for products sold in online retail settings. For each of these Australian labels (with the exception of country-of-origin), formal guidance material does not appear to have considered the online environment as yet. International work on this issue is also not yet settled [11], though draft guidelines due for consideration by the Codex Committee on Food Labelling in May 2023 support the display of relevant product information on product webpages prior to online retail sale [12].

This issue is becoming increasingly salient given the considerable growth in online food retail in many high- and middle-income countries, particularly since the beginning of the COVID-19 pandemic [13]; in Australia, in 2022, it has been estimated that almost half of consumers do at least some grocery shopping online [14]. However, there have been few in-depth investigations of the extent to which product labelling is applied in online food retail environments [15].

The primary aims of the current study were to assess, on food product webpages in online supermarkets in Australia, 1) the prevalence of mandated product information across supermarkets and by supermarket, 2) the prevalence of voluntary HSR labels and whether such labels were differentially applied to healthy and unhealthy products (as has been found for HSR uptake on physical food packaging [16]), across supermarkets and by supermarket, and 3) whether the prevalence of immediately visible labels (v labels only visible following further action e.g. scrolling, clicking) differed by supermarket, as the online setting offers different opportunities for highlighting or minimising product information, including via the specific architecture of each site/page. As secondary outcomes, we also assessed differences in labelling prevalence according to whether a product was private label (i.e. the supermarket’s own/generic/ “home” product line) or branded, as retailers are more likely to possess information about their own products, which may have implications for a (future) duty to display the relevant information in this environment.

## Methods

### Data collection

**Fig. 1:**
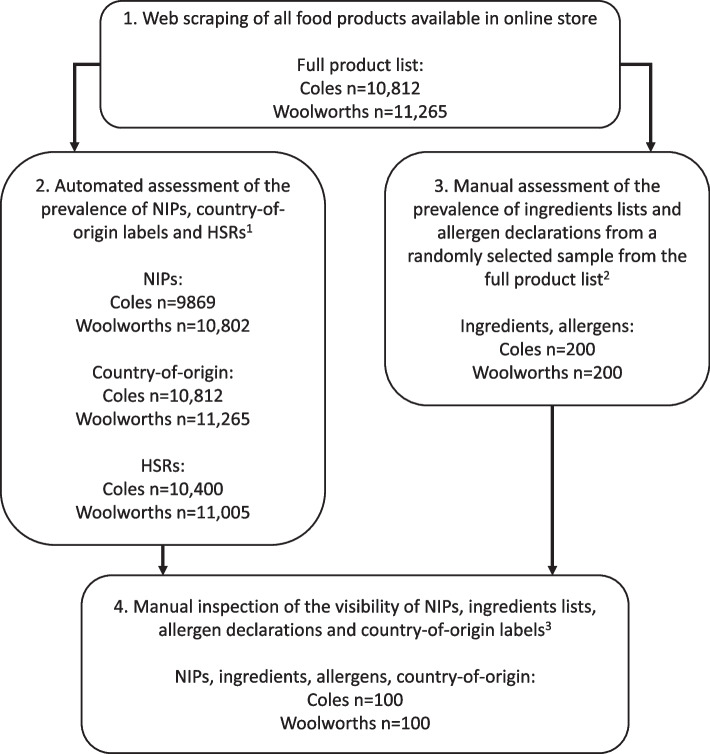
Data collected from two online supermarkets in Australia in April–May 2022 and outcomes assessed. NIP = nutrition information panel, HSR = Health Star Rating. 1 After excluding ineligible food products. Excluded products – NIPs: herbs, spices, vinegar, salt, tea, coffee, raw produce, gelatine, pectin and water; country-of-origin: none; HSRs: baby formula, food for infants, formulated meal replacements, formulated supplementary foods and formulated supplementary sports foods. 2 After excluding ineligible food products. Excluded products – ingredients: after excluding water and single ingredient products that do not provide an ingredients list; allergens: products that do not contain allergens. 3 From a randomly selected sample of foods products displaying relevant label

This study used two approaches to collect data from the online stores of the two leading supermarket retailers in Australia, Woolworths and Coles. These two supermarkets together account for approximately two-thirds of the total market share [17]; the next largest supermarket, Aldi, has less than 10% of total market share and does not offer online grocery shopping. See Fig. 1 for a summary of data collection and outcomes assessed. No ethics approvals were required as our study did not involve the use of any animal or human data, as per both institutional and IJBNPA policies.

Firstly, automated web scraping was performed using bespoke C +  + coding in April–May 2022, which collected page URL and product name and barcode from all individual food and beverage product pages. Where available, the presence of NIPs, country-of-origin labelling and HSRs was also recorded through the web scrape. Products with multiple packaging sizes on a supermarket’s store were all included for analyses (i.e. each different size was included in the analysis and counted as individual products). This dataset was used to assess the presence of three labels (NIP, country-of-origin and HSR).

A manual data collection was also conducted to assess the presence of ingredients lists and allergen declarations and the visibility of all five labels (NIP, ingredients, allergens, country-of-origin and HSR), as this could not be adequately assessed via the automated collection. Two researchers (DM, LS) created and tested a common protocol for both supermarkets and independently collected data. A sample of products was derived by assigning a random number against each product in the full product list scraped from the supermarkets’ websites, generated using the “randbetween()” function in Microsoft Excel. The random numbers were then sorted from smallest to largest and the top results were selected. For the assessment of labelling presence, any products ineligible to carry the label (according to the relevant label’s requirements [5–7]) were replaced with the next eligible product in the sorted list. For the assessment of visibility, pages not displaying the label were replaced with the next product displaying the label in the sorted list (i.e. all product pages checked for visibility provided the relevant information in some form). The replacement was necessary as it was not possible to account for eligibility in the randomisation process. This randomisation and manual data collection was conducted seven times in total, once for each of the outcomes being assessed manually, for each supermarket. To assess presence of ingredient lists and allergen declarations, we collected 200 products from each supermarket for each outcome (i.e. *n* = 400 in total for each outcome), and for visibility of each label *n* = 100 products were assessed for each supermarket for each outcome (i.e. *n* = 200 in total for each outcome). For each outcome, the proportion of products assessed across categories (as per categorisation in the FoodSwitch database; see below) was comparable (maximum ± 5 percentage points) between supermarkets.

### Obtaining additional product information for food products identified online

To complement labelling information obtained online, products captured from the online data scrape were matched to entries in the Australian FoodSwitch database to obtain additional product information. This database consists of detailed product information for > 100,000 individual products available in Australian supermarkets. In brief, FoodSwitch data is collected in two different ways: firstly, through an annual in-store collection by a trained team from major supermarkets in Australia; and secondly, through crowd-sourcing by consumers using a downloadable FoodSwitch app. A data processing team then completes data input and cleaning and product categorisation. A fuller description of data collection and processing is available elsewhere [18, 19]. Recent analysis suggests that the FoodSwitch database contains information for > 95% of packaged products purchased from supermarkets in Australia [20].

Products collected from the online stores by web scraping were matched to the additional product information in the FoodSwitch database by their unique barcodes, or by product name and other descriptors if barcodes were not available. The additional product attributes that were available in the FoodSwitch database included full NIP, ingredient and allergen information, HSR and product branding for all products.

Products were subsequently categorised as “private label” (i.e. owned and manufactured by a retailer, or produced and/or packaged solely for a retailer) according to public lists provided by Woolworths and Coles at the time of data collection [21, 22]. Products which were not private label (i.e. produced by and packaged for an independent company) were categorised as “branded”.

### Study outcomes

#### Presence of mandatory food labelling

Labels were recorded as “present” or “not present”. To be considered present for the purposes of this study, the information must have been available on the individual product page and met relevant requirements that currently apply to on-pack labelling:

- NIP: must at a minimum detail the serving size and number of servings per pack, as well as the energy, protein, total carbohydrate, total sugars, total fat, saturated fat and sodium content per 100 g/mL and serving, including appropriate units [5]. Compliance against contingent requirements (e.g. to list other nutrients if a health or nutrition claim is made) were not assessed.
- Ingredients: products must provide a statement that lists each ingredient in the product in descending order of weight using common, generic or descriptive names [6]. Allergens must also be clearly identified using generic terms and highlighted in the ingredients list [7].
- Allergens: the presence of allergens must also be separately detailed in generic terms. In addition, a broader list of declarations and statements must be displayed if the product contains, or does not contain, certain ingredients [7].
- Country-of-origin: all packaged and non-packaged food sold at retail outlets must carry a standard mark and/or a text statement denoting the location of production, processing and/or packing. The appropriate label depends upon the type of product, the product packaging and the location of the various processes claimed [8].

Certain categories of products are exempt from the requirements for each label type [5–7] (except country-of-origin, which is required for all foods) and were therefore excluded from the relevant analyses (Fig. 1).

#### Display of voluntary food labelling

The HSR is an interpretive summary spectrum-type FOP nutrition label used in Australia and New Zealand. Scores range from 0.5 to 5 HSRs, with higher HSRs indicating healthier products. A product page reporting a HSR in any form, including as text, was considered to be displaying a HSR. Products that are ineligible to display a HSR were excluded from analyses [9]. HSRs were recorded as “present” or “not present”.

We then assessed whether the online display of HSRs differed according to product healthiness. Products were categorised as “healthy” if they had a HSR ≥ 3.5 and were considered “unhealthy” if they had a HSR < 3.5, based on previous government and independent research and policy [23–26]. HSRs were calculated using product information available in the FoodSwitch database [18].

#### Visibility of food labelling

**Fig. 2:**
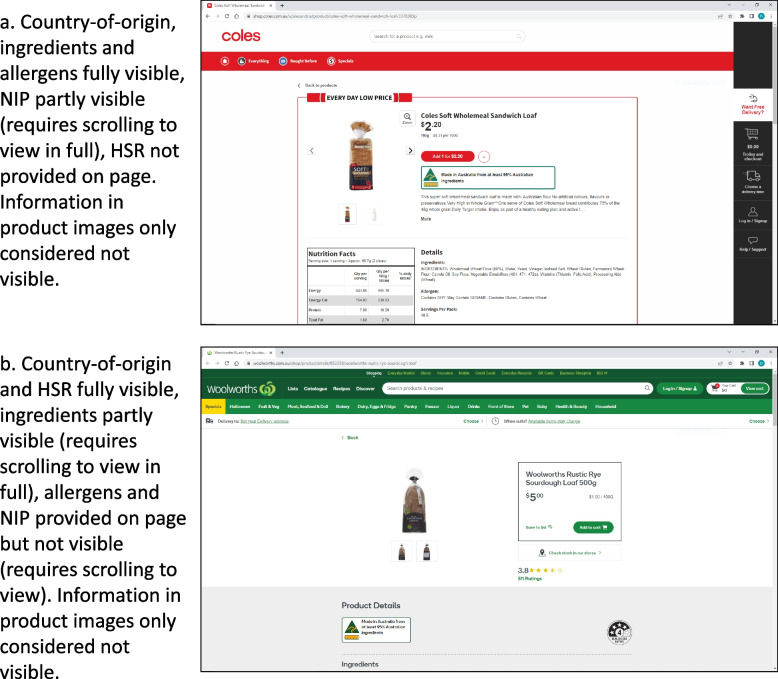
Example of assessment of visibility. NIP = nutrition information panel, HSR = Health Star Rating

If the relevant labels were immediately observable in part or in whole on the individual product page without scrolling, clicking or expanding sections it was recorded as visible (as illustrated in Fig. 2). If no part of a label was visible it was recorded as not visible. Product information only available in product images is more difficult to access, particularly for vision-impaired people, thus was not recorded as visible.

Visibility was assessed using the Google Chrome browser, with medium font size and 100% zoom and maximised to fit screen, on a single monitor displayed in 1920 × 1080 resolution in landscape.

### Analyses

The proportion (%) of product pages displaying each labelling type is reported in total, separately for each supermarket and by product branding (private label vs. branded). The prevalence of HSRs is further reported by product healthiness (< 3.5 HSR vs. ≥ 3.5 HSR). The visibility of labelling is reported as proportion visible for each supermarket.

Differences in proportions displaying each label between supermarkets, between less and more healthy products displaying HSR within each supermarket, and in the visibility of labels between supermarkets were compared using Pearson chi-squared tests. We adjusted p-values for multiple testing by controlling the familywise error rate using the Hochberg method [27], which is more powerful but less conservative than other similar techniques [28]; we elected to control the familywise error rate (i.e. the probability of any Type I errors), rather than the false discovery rate (i.e. the expected proportion of Type I errors), as a more stringent adjustment and given the relatively small number of comparisons planned [29]. All statistical testing was conducted using RStudio 2023.06.0 + 421. A conventional significance level of two-sided α = 0.05 was used. We decided a priori not to test differences in the secondary outcome of interest (display of labels by private label and branded products) as this analysis was exploratory in nature.

## Results

A total of *n* = 10,812 products were identified through data scraping from Coles and *n* = 11,265 products from Woolworths (Fig. 1). This represents 100% of the eligible food products available in the respective online stores at the time of data collection.

### Mandatory food labelling

Across both supermarkets, the provision of NIPs (49%), ingredients lists (34%) and allergen declarations (53%) was largely incomplete, though almost all products, across both supermarkets, displayed country-of-origin labels (93%) (Table 1). Woolworths was more likely to provide NIPs (59% v 37%, *p* < 0.001) and allergen declarations (61% v 46%, *p* = 0.010), while Coles was more likely to display country-of-origin labelling (94% v 92%, *p* < 0.001).

**Table 1:** Presence of mandatory labels for food products for two online supermarkets in Australia, 2022. NIP = nutrition information panel

Label	All products	Coles	Woolworths	Difference in presence of label between supermarkets,* p*-value
	n displaying label/total n assessed (% displaying label)			
**NIP**	10,082/20671 (49%)	3665/9869 (37%)	6417/10802 (59%)	*p *< 0.001
**Ingredients**	134/400 (34%)	61/200 (31%)	73/200 (37%)	*p* = 0.244
**Allergens**	213/400 (53%)	92/200 (46%)	121/200 (61%)	*p* = 0.010
**Country-of-origin**	20,519/22077 (93%)	10,196/10812 (94%)	10,323/11265 (92%)	*p* < 0.001

### Health Star Ratings

**Fig. 3:**
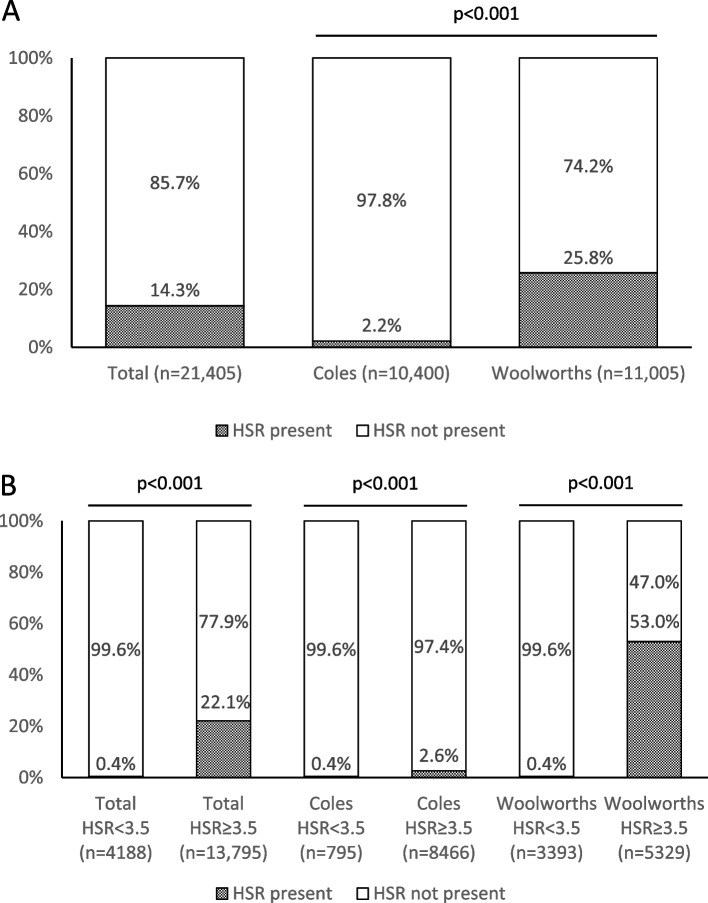
Proportion of food product pages providing HSRs for two online supermarkets in Australia, 2022. HSR = Health Star Rating. Panel A shows the presence of HSRs in total and for each supermarket overall. Panel B shows presence of HSRs for products eligible to display a HSR < 3.5 and for products eligible to display a HSR ≥ 3.5, by supermarket. To determine the HSRs that products were eligible to display, food products collected from online stores were matched using their barcodes or other identifying information to FoodSwitch data. 89% of products from Coles and 79% of products from Woolworths were matched and had HSR information available

The display of HSRs was overall low (14% across both supermarkets), though more likely for Woolworths than Coles (26% v 2%, *p* < 0.001) (Fig. 3). HSRs were more likely to be displayed on higher-scoring products (0.4% of products scoring < 3.5 HSR v 22% of products scoring ≥ 3.5 HSR, *p* < 0.001), with the difference more marked for Woolworths (0.4% of products scoring < 3.5 HSR v 53% of products scoring ≥ 3.5 HSR, *p* < 0.001) than Coles (0.4% of products scoring < 3.5 HSR v 3% of products scoring ≥ 3.5 HSR, *p* < 0.001), partly reflecting the overall higher prevalence of HSR labelling for Woolworths.

### Visibility of food labelling

**Fig. 4:**
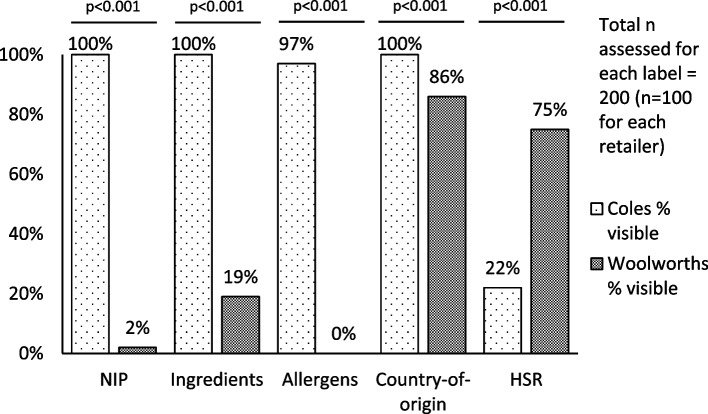
Visibility of labels on food product pages providing the relevant information anywhere on page for two online supermarkets in Australia, 2022. NIP = nutrition information panel, HSR = Health Star Rating

Across products making the relevant information available somewhere on the product page, Coles made NIPs and ingredients and allergen information immediately visible on all or almost all product pages, whereas for Woolworths visibility of these labels was far less complete (NIPs 100% v 2%, *p* < 0.001; ingredients 100% v 19%, *p* < 0.001; allergens 97% v 0%, *p* < 0.001) (Fig. 4). Coles also made country-of-origin information visible for all products, though Woolworths also made this information readily visible on most pages (100% v 86%, *p* < 0.001). However, less than one-quarter of HSRs were visible for Coles, while Woolworths made most HSRs immediately visible (22% v 75%, *p* < 0.001).

### Presence of labels for private vs. branded food products

See Additional File 1 for results.

## Discussion

Consumers are increasingly shifting to online grocery settings, which poses new issues for the display of information about food products that supports decision-making. In our study, which to the best of our knowledge is the first to comprehensively assess the completeness of mandatory and voluntary labelling in online supermarkets in Australia, we found that country-of-origin labelling is almost complete**,** whereas nutrition, ingredient and allergen information were much less frequently displayed. HSRs, an easy-to-use guide to the overall healthiness of a product, are rarely made available and there was evidence, across both supermarkets, of their selective application to products that would receive higher scores. Overall, each supermarket differed distinctly in how much labelling information was made available online, with Woolworths more likely to provide NIPs, allergen declarations and HSRs, while Coles was marginally more likely to provide country-of-origin labelling. The design of each supermarket’s product pages also resulted in marked differences in labelling visibility (where such information is provided in the first place), with NIPs, ingredients lists and allergen declarations much more readily apparent on Coles’ website, Woolworths making HSRs more visible on their website, and both supermarkets displaying country-of-origin information prominently.

NIPs, ingredients lists and allergen declarations provide useful and potentially critical information to consumers and regulators. For consumers in particular, missing information poses health and safety risks in terms of both acute adverse reactions and non-communicable diseases. The inconsistent online display of these labels, which are mandated on physical product packaging to manage such risks, is cause for concern. In addition, our observation that country-of-origin labels are more frequently present than other mandatory labels and that HSRs are far more likely to be displayed for higher-scoring products suggests that certain label types may be employed as marketing tools, the potential for which has been identified by the World Health Organization [1]. That is, country-of-origin labels and high HSRs may offer some appeal to consumers and thus more readily be applied and/or prominently displayed to influence purchases. The finding regarding country-of-origin is particularly striking given they were only made mandatory in 2018, compared to, for example, 2002 for NIPs.

The availability of HSRs on-pack has been subject to regular monitoring in recent years. A 2019 evaluation, five years into the implementation of the system, found that HSRs were displayed on 41% of eligible products in-store, with the two supermarkets assessed in our study applying HSRs to around 9 in 10 of their own products [16]. Uptake on physical products has likely further improved since then, leaving the online display of these easy-to-use labels lagging even further behind. There was evidence that the label was being selectively applied on pack to higher-scoring products overall [16], as also found in our study, lending credence to concerns that HSRs may be applied for marketing purposes [30]. Given that the two supermarkets assessed in our study were already applying HSRs to most of their own products in-store several years ago and retailers presumably have better access to such proprietary information, the lack of near-complete HSR information for private label products online (particularly for Coles) is notable. Additionally, one supermarket (Woolworths) provides an occasional swap function based on HSRs in their online store, though implicated products do not necessarily display a HSR on physical packaging and/or online. This suggests that information on HSRs is available to the retailer but not presented to users. Our findings of low uptake overall and selective application to higher-scoring products provide added support to consistent calls made by public health and consumer researchers and advocates to make the HSR system mandatory [31–35]. Our study further highlights another key gap in the application of these important tools for addressing diet-related disease.

The agency responsible for administering Australia and New Zealand’s joint food regulatory system, Food Standards Australia New Zealand, is tasked with developing and reviewing regulatory measures that protect public health and safety and support informed consumer decision-making (as well as prevent misleading or deceptive conduct) [36]. Amidst a rapidly changing food retail environment, our findings suggest that current regulations do not adequately support either objective in the online setting. These results highlight the need for national and international standards to be updated to require that equivalent or comparable information be provided for all products available online prior to purchase. The current reference to online provision of information for country-of-origin labelling, which provides that only the physical packaging of products sold online must comply with requirements [8], is insufficient as the consumer must necessarily have already purchased and received the product, reversing the logical timing of the labelling intervention and nullifying its potential to enable informed decision making. Future considerations for product labelling in online settings may also need to define roles and responsibilities at the regulatory level (who is formally charged with displaying such information?) and on a practical basis (who has the information that is to be displayed, where and how should be it displayed?), as in the online space the burden of providing product information would likely shift from the manufacturer, distributor and/or importer to the retailer. Further regard must also be given to the need to update multiple sources of information (physical packaging, product image/s online, product information online) when a product is reformulated to minimise discrepancies, acknowledging that some lag is likely. Grocery aggregators which compile information from multiple retailers, such as Frugl, DoorDash and YourGrocer, may also be implicated. Of some additional concern is that online store users may bypass individual product pages entirely by adding products to cart directly from home, specials or category pages and user- or retailer-set lists, which usually do not provide any information about a product beyond an image, a name, a price and a specials flag.

Revised standards which account for online settings will also need to prescribe the format of the online display, given issues with visibility identified in our study. The online architecture is more modifiable than physical product packaging and, unlike in physical settings, the consumer view of the product page can change dramatically from user to user depending on hardware, software, user customisation and retailer personalisation. The current study used inclusive criteria to assess visibility by allowing even marginally visible labels to be counted as present; in practice far fewer labels would likely be seen and understood by consumers, particularly when accessing the online store via mobile phone. Regardless, the diverging results seen in our study, where one supermarket made almost all labels (where provided) visible immediately while the other tended to only initially display country-of-origin and HSR, shows that it is possible for online supermarkets to make information readily apparent to users. Furthermore, relying on product images on a product page is not supported as these are less accessible to consumers (e.g. with vision impairment or poor internet access) and often do not provide views of all sides of a product and/or are not provided in sufficient resolution to view the required information. Of note, however, is that all the information manually assessed in our study, when provided on the product page, was clear and legible.

Despite the lack of explicit direction on the provision of product information in online retail settings, the guiding principle presumably remains the same, i.e. information about a product should be available to consumers alongside the product in a retail environment prior to purchase, and this information should be displayed both consistently and in a manner compliant with relevant regulation or guidance. As such, in the interim we recommend that retailers apply all relevant labels to eligible products available online, including to match their proven commitment to displaying HSRs on private label products in-store, acknowledging that branded products may require some additional liaison with other companies regarding the provision of relevant information. This would demonstrate an evolving understanding of corporate social responsibility by retailers and may be of interest to investors that prioritise environmental, social and governance considerations [37, 38]. Regardless, with any display of product information online retailers should pay heed to completeness, accuracy and consistency, while carefully considering how such information is presented to consumers to better support decision-making.

Few studies have assessed the prevalence of product information online, with diverse methodologies leading to varied results even within countries at similar time points. Most such studies have been conducted in high-income countries and used smaller (generally n < 1000 products) and/or intentionally selected samples of products [39–47]. Notably, these studies have found that the display of NIPs, ingredients and allergen information and FOP nutrition labelling in online supermarkets is inconsistent and largely far from complete, while those labels which are available are mostly and occasionally entirely hidden from view. One Hong Kong-based study of product information online (*n* = 22,365), similar to ours, found that ingredients information was available for 30% of products and NIPs for 20% of products overall, with half of the supermarkets surveyed providing this information on no or very few products [48]. Our study builds on and extends these prior studies by drawing upon a large sample of products to investigate the presence and visibility of a comprehensive list of various labels and assess the differential application of voluntary FOP nutrition labelling to higher/lower scoring products.

Studies on how consumers view and use information in online supermarkets are even rarer. One notable UK-based study (*n* = 40 participants) tracked eye movements to assess how consumers navigated a real-world online store and looked at the information made available across the various screens presented [49]. Participants were found to click through to individual product pages 65% of the time but only spend, on average, approximately 12% of their time on that page looking at nutrition, ingredients, allergen or FOP nutrition labelling in total. However, it is not known how often this information was made available on product pages, nor whether this information was immediately visible to the participant or required further action.

### Future research directions

The great diversity in methods applied in the above studies, as well as the lack of data outside of a high-income country context, suggests that a standardised and accessible protocol for assessing the completeness of product labelling online will help to facilitate comparisons between supermarkets, locations and time points. In particular, repeat assessments using comparable methods are critical to monitor progress in the adoption of relevant labelling online. Future quantitative and qualitative research is also required to understand how consumers shop for groceries online, and view and use product information in this environment. An exploration of how and why retailers make information available on their online stores (or not), retailer policies around nutrition labelling, and potential mechanisms to support informed consumer decision-making in this setting would also facilitate efforts to improve practices.

### Strengths and limitations of the current study

Strengths of our study include the large number of products collected from the two dominant supermarkets in Australia, which includes the entire product range available at a point in time. This permitted a comprehensive analysis of the full situation as relevant to the majority of online grocery shoppers in Australia at that time. While we conducted statistical testing across multiple (*n* = 13) comparisons, we also applied a statistical adjustment to control the chances of returning false results; regardless, the magnitude of the differences observed remains of primary importance.

Limitations include, conversely, the use of data collected at a single point in time and from two supermarkets only; given the nature of online environments there is potential for the situation to change rapidly and our results may not be representative of all online supermarket retailers in Australia. The automated data collection may not capture all relevant information accurately, although no errors, discrepancies or omissions were identified during manual checks. While efforts were made to standardise the manual data collection process, human error and subjectivity could have introduced inconsistencies or bias into the results. Finally, as noted previously, we used a pre-determined and consistent protocol to assess visibility and any change in format and display, particularly to tablet- or phone-based browsing, would have changed results.

## Conclusion

While information on product composition is largely lacking in online supermarkets, country-of-origin labelling is comprehensive. HSRs were infrequently displayed overall and more likely to be provided for higher-scoring products. Differences in website architecture led to diverging results in the visibility of product information on product pages, when made available.

Missing or difficult to find information about product composition is not just an issue for consumer choice, but also poses potentially serious health risks. Ingredient and particularly allergen information is critical to immediate safety from adverse reactions, while the provision of nutrition information is a key tool to combat risks from non-communicable diseases as well as overweight and obesity. Our findings highlight the need for regulation to be updated and strengthened to better account for the increasingly important online retail food environment.

### Supplementary Information

**Additional file 1:** **Supplementary Table 1. **Proportion of food product pages displaying mandatory labels and Health Star Ratings in total, by private label and by branded products, for two online supermarkets in Australia, 2022. NIP = nutrition information panel, HSR = Health Star Rating.

**Additional file 2.** STROBE Statement—Checklist of items that should be included in reports of cross-sectional studies.

## Data Availability

The datasets used and/or analysed during the current study are available from the corresponding author on reasonable request.

## Abbreviations

FOP: Front-of-pack
NIP: Nutrition information panel
HSR: Health Star Rating
