# Supplementary material for: Current food labelling practices in online supermarkets in Australia
Source: Int J Behav Nutr Phys Act. 2023 Sep 26;20:105. doi: 10.1186/s12966-023-01504-3 (PMC10521447; doi:10.1186/s12966-023-01504-3)
Supplement: Supplementary file 1 — Additional file 1: Supplementary Table 1. Proportion of food product pages displaying mandatory labels and Health Star Ratings in total, by private label and by branded products, for two online supermarkets in Australia, 2022. NIP = nutrition information panel, HSR = Health Star Rating. [file 12966_2023_1504_MOESM1_ESM.docx]

### *Presence of labels for private vs. branded food products*

For Coles, private label products were more likely to provide ingredients (private vs. branded, 48% v 25%), allergen (59% v 43%) and country-of-origin (100% v 93%) information than branded products, with branded products more likely to display NIPs (1% v 46%) (**Supplementary Table 1**). For Woolworths, allergen (private v branded, 65% v 59%) and country-of-origin (94% v 91%) information were more common for private label products than branded products, while NIPs (52% v 64%) and ingredients (30% v 38%) were more commonly displayed for branded products than private label products. Woolworths displayed HSRs more often for private label products than branded products (private v branded, 62% v 17%), however the opposite was true for Coles (0.04% v 3%).

**Supplementary Table 1. Proportion of food product pages displaying mandatory labels and Health Star Ratings in total, by private label and by branded products, for two online supermarkets in Australia, 2022.** NIP = nutrition information panel, HSR = Health Star Rating.

| **Label** | **Coles** | | | **Woolworths** | | |
| --- | --- | --- | --- | --- | --- | --- |
|  | **n displaying label/total n assessed (% displaying label)** | | | **n displaying label/total n assessed (% displaying label)** | | |
|  | **All products** | **Private label** | **Branded** | **All products** | **Private label** | **Branded** |
| **NIP** | 3665/9869 (37%) | 25/1997 (1%) | 3640/7872 (46%) | 6417/10802 (59%) | 1012/1963 (52%) | 5405/8433 (64%) |
| **Ingredients** | 61/200 (31%) | 24/50 (48%) | 37/150 (25%) | 73/200 (37%) | 12/40 (30%) | 61/160 (38%) |
| **Allergens** | 92/200 (46%) | 23/39 (59%) | 69/161 (43%) | 121/200 (61%) | 30/46 (65%) | 91/154 (59%) |
| **Country-of-origin** | 10196/10812 (94%) | 2374/2379 (100%) | 7822/8433 (93%) | 10323/11265 (92%) | 1973/2092 (94%) | 8350/9173 (91%) |
| **HSR** | 224/10400 (2%) | 1/2337 (0%) | 223/8063 (3%) | 2835/11005 (26%) | 1292/2069 (62%) | 1543/8936 (17%) |
| HSR<3.5 | 3/795 (0%) | 0/135 (0%) | 3/660 (0%) | 12/3393 (0%) | 0/108 (0%) | 12/3285 (0%) |
| HSR≥3.5 | 221/8466 (3%) | 1/1878 (0%) | 220/6588 (3%) | 2823/5329 (53%) | 1292/1715 (75%) | 1531/3614 (42%) |

Note: no significance testing undertaken, see Methods for further information
